# Biochar Addition Reduces the Effect of High Nitrogen on Soil–Microbial Stoichiometric Imbalance in Abandoned Grassland on the Loess Plateau of China

**DOI:** 10.1002/ece3.70875

**Published:** 2025-01-30

**Authors:** Shuainan Liu, Mingjun Xie, Wende Lu, Xinyue Zhang, Mengyin Du, Yao Yao, Jianyu Yuan, Guang Li

**Affiliations:** ^1^ College of Forestry Gansu Agricultural University Lanzhou China; ^2^ College of Grasslands Gansu Agricultural University Lanzhou China

**Keywords:** abandoned grassland, biochar amendment, high nitrogen, loess plateau, soil–microbial biomass, stoichiometric imbalance

## Abstract

Progressively higher atmospheric nitrogen (N) deposition increasingly affects soil ecosystems' elemental cycling and stability. Biochar (BC) amendment has emerged as a possible means of preserving soil system stability. Nevertheless, the pattern of soil–microbial nutrient cycling and system stability in response to BC after high N deposition in ecologically sensitive regions remains uncertain. Therefore, we investigated the effects of high N (9 g N·m^−2^·a^−1^), BC (0, 20, 40 t·ha^−1^), and combinations of the treatments on soil organic carbon (SOC), total nitrogen (TN), total phosphorus (TP), microbial biomass carbon (MBC), nitrogen (MBN), phosphorus (MBP), microbial entropy (*q*_MB_), and stoichiometric imbalance (C_imb_:N_imb_:P_imb_). We found that high N addition decreased topsoil (0–20 cm) TP, C:N, *q*_MBN_, and C_imb_:N_imb_ values and increased TN, C:P, N:P, *q*_MBP_, C_imb_:P_imb_, and N_imb_:P_imb_ values. However, BC addition increased 0–40 cm soil *q*_MBC_ and N_imb_:P_imb_ values and decreased *q*_MBN_, C_imb_:N_imb_, and C_imb_:P_imb_ values. Meanwhile, high BC additions attenuated BC's promotion of soil–microbial nutrients. We observed that a mixture of high N and BC increased the 0–40 cm SOC and TP content, promoted the accumulation of MBN and MBP in the subsoil (20–40 cm), and decreased the topsoil C_imb_:P_imb_ and N_imb_:P_imb_ values compared to high N additions. The impact of high N and BC additions on N and P elements varied significantly between the different soil depths. In addition, redundancy analysis identified C:N, MBC, MBN, and C:P as pivotal factors affecting alterations in soil *q*_MB_ and stoichiometric imbalance. Overall, adding BC reduced the negative impacts of high N deposition on the stability of soil–microbial systems in the Loess Plateau, suggesting a new approach for managing ecologically fragile areas.

## Graphical abstract

High N and biochar additions affected soil nutrients and microbial biomass differently, leading to an imbalance in their stoichiometry. High biochar additions diminished the contribution of biochar to soil nutrients and microbial biomass. Biochar addition can offset some of the negative effects of high N deposition on soil nutrients, microbial biomass, and stoichiometric imbalances between them in the Loess Plateau.

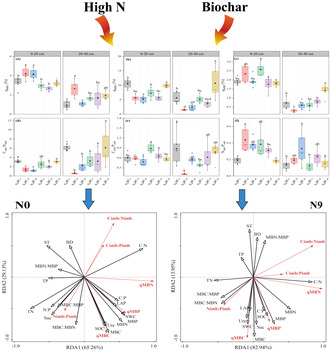

## 1 Introduction

Soil plays a crucial role in controlling the supply and circulation of nutrients in terrestrial ecosystems, and the levels of key nutrients like carbon (C), nitrogen (N), and phosphorus (P) can dramatically influence the elemental cycling and stability of an ecosystem (Zhao et al. 2021; Pandey et al. 2024). At the same time, soils harbor the most diverse microbial communities on Earth, and the microorganisms within these communities can, directly and indirectly, drive essential functions such as nutrient cycling in ecosystems through processes that include oxidation, reduction, and decomposition (Hartmann and Six 2022; Chi et al. 2023). Previous studies have demonstrated that soil microbial biomass carbon (MBC), nitrogen (MBN), and phosphorus (MBP) correspond to the active components of microorganisms (Li, Yu, and Song 2022; Manral et al. 2023). These active fractions play a vital role in soil element cycling and energy flow as key biological components in soil ecosystems (Zhao et al. 2021; Dong et al. 2022). In addition, microbial entropy (*q*_MB_) is often considered as the proportion of microbial biomass within soil base nutrients, and an increase in *q*_MB_ can indicate a better accumulation of soil nutrients (Xie et al. 2023). Prior research has demonstrated that *q*_MB_ reflects the microbial biomass that a unit of resource can support, effectively capturing variation in soil nutrients and nutrient utilization efficiency (Chi et al. 2023; Xie et al. 2023). Therefore, exploring the characteristics of changes in soil nutrients and microbial biomass can provide a deeper understanding of soil health and nutrient cycling processes in ecosystems.

Recently, researchers have often focused on the interactions and equilibrium relationships of multiple chemical elements in different ecosystem components (e.g., soils and microorganisms) and ecological processes through ecological chemometrics (Gao et al. 2022; Chi et al. 2023). Multiple studies have indicated that the stoichiometric relationship between soil nutrients and microbial biomass is an important tool for studying the cycling and limiting effects of soil nutrients and can effectively characterize soil texture, the degree of nutrient demand, and resource use strategies (Gao et al. 2022; Liang et al. 2023). Research has demonstrated that changes in soil and microbial biomass stoichiometry ratios may strongly affect ecosystem structure, function, and processes (Chi et al. 2023; Qin et al. 2024). In addition, when the stoichiometry of soil supply does not match the needs of microorganisms, the growth metabolism of soil microorganisms will be limited by soil‐specific nutrients (i.e., stoichiometric imbalance) (Chi et al. 2023; Xie et al. 2023). However, the stoichiometric patterns among soil–microbial systems are susceptible to the combined effects of climate, land use type, soil type, and other factors, leading to changes in the stoichiometric ratios and their associated system stability (Yuan et al. 2019; Liang et al. 2023; Ren et al. 2024). Therefore, a better understanding of the stoichiometric relationship between soil and microbial biomass is of great importance in identifying the mechanism of their synergistic regulation of the dynamic balance among ecosystem nutrients.

In recent decades, atmospheric N deposition has become an important issue in global climate change owing to human agricultural activities and fossil fuel combustion (Jing and Wang 2020). Global N deposition is reportedly expected to exceed the global N critical load by 2050 (195 Tg N·year^−1^) (Li et al. 2020). Additionally, as one of the top three N deposition hotspots in the world, China's N deposition has locally reached as high as 30–50 kg N·ha^−1^·year^−1^, approaching the values observed in natural ecosystems (Jiang et al. 2024). Research has indicated that progressively higher N deposition can reduce soil microbial biomass, microbial entropy values, and nutrient effectiveness by altering soil microbial community structure and activity (Hu et al. 2023; Yang et al. 2023). This can also further alter the stoichiometric patterns of soil systems (Gong, Zhang, and Guo 2019; Han et al. 2019) and affect soil nutrient balance and system stability (Xiao et al. 2021b). Consequently, controlling the stoichiometric pattern of soil under high N deposition by developing effective remediation measures to stabilize ecosystems is one of the critical research issues both within China and worldwide.

Biochar (BC) is a solid substance rich in inert carbon produced by pyrolysis (under anaerobic or low‐oxygen conditions) of biomass such as crop straw or urban waste (Rombola et al. 2022). It is widely applied to fields for the purpose of environmental remediation owing to its low cost and high availability, as well as its high chemical stability and richness in surface functional groups (Yan et al. 2022). In general, BC addition improves soil structure (Sarauer and Coleman 2023) and effectively increases soil total organic matter, available nutrients, and pH (Karimi et al. 2019; Li et al. 2020; Khadem et al. 2021), thus contributing to the soil's ability to retain water and fertilizer and nutrient effectiveness (Rehman et al. 2020; Zhao et al. 2023). Multiple studies have shown that the well‐developed pore structure of the BC surface provides a microhabitat for predators in the soil (Li et al. 2020; Sarauer and Coleman 2023), thus altering the structural composition of some soil organisms, such as nematodes (Cole et al. 2021). However, the results of previous studies have not been consistent, likely because they have been influenced by single factors or combinations of factors such as climatic conditions, soil texture, type of feedstock, rate of addition, and application rate in the study area (Karimi et al. 2019; Li et al. 2020; Khadem et al. 2021). For instance, BC addition may promote (Song et al. 2019), inhibit (Guo et al. 2020; Li et al. 2020), or have no effect (Sarauer and Coleman 2023) on soil microbial activity. On the one hand, BC addition has been shown to improve the microbial community composition by increasing the effective nutrients in the soil, increasing the relative abundance of nitrogen‐fixing and phosphorus‐dissolving bacteria, among others (Hu et al. 2023; Zhao et al. 2023), and increasing enzyme activity and microbial biomass (Karimi et al. 2019; Song et al. 2019). However, it has also been shown that higher pyrolysis temperatures in BC preparation may produce more alkaline compounds and reduce the availability of unstable nutrients for soil microorganisms (Guo et al. 2020). Meanwhile, when BC is added in excessive amounts, it can disrupt the optimal environment for soil microbial growth and inhibit microbial activity by reducing the growth efficiency of soil microbes (Zhang et al. 2022c; Zou et al. 2023). In addition, BC additions can lead to changes in ecosystem stoichiometry patterns. Several studies have found that BC addition increased soil stoichiometric ratios by enhancing soil nutrient concentrations (Chang et al. 2022; Zhang et al. 2023) and decreased C:P and N:P ratios of plants (Zhang et al. 2023). This change has also resulted in altered nutrient utilization strategies and microbial entropy values of soil microorganisms (Guo et al. 2020), leading to imbalances in the stoichiometric pattern of the soil system, which in turn affects the microbial growth utilization efficiency and microbial response to the external environment (He, Yan, and Fan 2023). Collectively, these previous studies have shown, to some extent, that BC plays a key role in improving quality and stabilizing the soil system. However, we have little understanding of how the stoichiometric relationship of soil systems under the addition of BC responds to future high N deposition.

The Loess Plateau region has extremely serious soil erosion problems and is a fragile ecological environment in China, characterized by the rapidity and sensitivity of its response to climate change (Liu and Wang 2021). In this area, ecological initiatives like converting agricultural land back into forest or grassland have been implemented recently and shown high potential (Liu et al. 2022). Recent reports have indicated that abandoned grassland (i.e., naturally restored grassland after the abandonment of cultivation) is a land use type that has accounted for a larger area on the Loess Plateau in recent years and can yield greater ecological benefits compared to plantation forest restoration (Tuo et al. 2018). However, based on a synthesis of previous studies, it was found that research targeting the ecological chemometrics in this area has mainly focused on the effects of the successional stage (Xiao et al. 2021a), years of vegetation restoration (Zhang et al. 2022b), vegetation type (Wang et al. 2022), and land‐use practices (Liu and Wang 2020) on soil, microbial biomass, and plant stoichiometry. Meanwhile, previous studies have also found that BC addition can improve the aggregate composition and stability of soils in the Loess Plateau (Yan et al. 2022), enhance the resistance of soils to rainfall erosion (Li et al. 2024), and, by altering the composition of bacterial communities in crop inter‐root soils (Li et al. 2023), effectively increase the water use efficiency of crops and crop yield (Hou et al. 2024). However, in general, this previous research neglected the stoichiometric relationship between soil and microbial biomass in abandoned grassland in response to BC addition under high N deposition, which seriously hinders a detailed understanding of soil nutrient cycling processes in the fragile habitats of the Loess Plateau.

A field experiment was conducted in the present study, implementing treatments with high N, BC, and combinations of the two amendments to investigate soil nutrient levels, microbial biomass, microbial entropy, stoichiometric ratios, and nutrient imbalances in grassland abandoned on the Loess Plateau. The present study aimed to answer the following two questions. (1) Does high N, BC, and their combined application affect changes in stoichiometric patterns between soil and microorganisms? (2) Does BC addition help reduce the effects of high N addition on soil nutrients, microbial biomass, and their stoichiometric balance? We posited the following hypotheses: (1) High N and BC addition could alter the nutrient interrelationships between soil and microbial biomass and affect the equilibrium of microbial biomass in terms of stoichiometric patterns; (2) BC addition can reduce the negative effects of high N on the nutrient and stoichiometric profiles of the soil and microbial biomass. These hypotheses help to enhance our understanding of restoration and conservation management strategies for fragile ecosystems and provide new perspectives for maintaining soil system stability.

## 2 Materials and Methods

### 2.1 Site Description

The research was conducted at the Institute of Water and Soil Conservation in Dingxi, Gansu Province, China (104°39′3″ E, 35°34′45″ N). The elevation of this region is between 2100 and 2250 above sea level, and it experiences a mesothermal semi‐arid climate (Liu et al. 2022; Xie et al. 2024). From 1998 to 2018, the average annual temperature of the study area was 6.3°C, with an average yearly precipitation of 386.70 mm, 52.7% occurring from July to September (Liu et al. 2022). The soil type is mainly entisol as described by the soil taxonomy of the USDA developed from loess parent material (Xie et al. 2024). In 1999, the region initiated a major project to enhance ecological management by converting farmland back into forests and grasslands, resulting in a notable increase in vegetation cover. Presently, the predominant vegetation types consist of artificially planted woodland and naturally regenerated secondary successional grassland. The initial properties of the 0–40 cm soil layer in the experimental area were 8.05 g·kg^−1^ organic carbon (OC), 0.41 g·kg^−1^ total nitrogen (TN), 0.79 g·kg^−1^ total phosphorus (TP), and pH 8.12. The dominant species of herbaceous vegetation were mainly *Stipa bungeana* Trin., *Leymus secalinus* (Georgi) Tzvelev., and *Artemisia frigida* Willd.

### 2.2 Experimental Design

In June 2022, we implemented a split–plot design for naturally restored abandoned grasslands with a consistent abandonment history (15 years or more) and homogeneous vegetation (all with about 80% vegetation cover). Three replicate sample plots were established in the experiment, with a 10‐m buffer strip between the replicates. In each replicated sample plot, two N addition levels (N_0_, 0 g N m^−2^ a^−1^; N_9_, 9 g N m^−2^ a^−1^) and three BC addition levels (BC_0_, 0 t ha^−1^; BC_20_, 20 t ha^−1^; BC_40_, 40 t ha^−1^) were applied according to a two‐factor randomized group trial, with a total of six treatment plots combined. The experiment had a total of 18 replicated treatment plots, each measuring 2 m × 2 m, with 1‐m buffer strips separating plots.

For high N addition (9 g N m^−2^ a^−1^) treatment, we refer to the background value of N deposition in the Loess Plateau in recent years (2.11 g N m^−2^ a^−1^) by expanding the localized N deposition by about 4.5‐fold to simulate the N control of future high N deposition on the soil system in the region (Jing and Wang 2020). Urea (CO(NH_2_)_2_) was dissolved in 2 L of distilled water and sprayed uniformly on the plots using a sprayer in June, August, and October 2022 and March, June, and September 2023, respectively (equivalent to dividing each year's N application into three equal parts along with an additional 1.5 mm of precipitation each year). The other treatments were sprayed with equal volumes of distilled water. Based on previous studies on BC addition (Yang et al. 2018; Li et al. 2020), in June 2022, we set three BC addition levels (BC_0_, BC_20_, BC_40_) within each N addition level. We applied them by tilling to mix BC into the 0–20 and 20–40 cm soil layers. The BC_0_ treatment was only excavated and backfilled without applying any material. BC was applied only in the first year and was not added in subsequent years. The raw material of this test BC (Henan Sanli New Energy Co. Ltd., Huixian, Henan, China) was wheat straw (pyrolyzed at 400°C), with the following compositional characteristics: OC 118.11 g kg^−1^, TN 7.67 g kg^−1^, TP 1.02 g kg^−1^, C:N 15.42, pH 9.36.

### 2.3 Sample Collection

Previous studies have shown that grassland plant roots are more distributed in the 0–40 cm soil layer (Wang et al. 2023b). Meanwhile, most of the microbial communities and their activities are restricted to the upper soil layers owing to the presence of the plant roots, apoplasm, and organic matter (Bargali et al. 2018; Padalia et al. 2018; Manral et al. 2020). Therefore, we collected samples of topsoil (0–20 cm deep) and subsoil (20–40 cm deep) in October 2023 by soil auger (20 mm in diameter) stratified in each replicate plot according to the “S” type five‐point sampling method. The collected samples were sifted through a 2‐mm soil mesh and then combined into a single sample, which was placed in a portable ice chest for transportation back to the laboratory for analysis of its various soil parameters. Simultaneously, a portable thermometer (JM628, Guangzhou Kunlun Automation Equipment Co. Ltd., Guangzhou, China) was employed to measure and document the subsoil temperature (ST) at 20 and 40 cm below the soil surface during the sampling process.

### 2.4 Measurement of Soil Indicators

Utilizing previously described research methods, we determined soil water content (SWC) (Liu et al. 2022), bulk density (BD) (Xie et al. 2024), sucrase (Suc), urease (Ure), and alkaline phosphatase (ALP) activities (Liu et al. 2022). Furthermore, soil organic carbon (SOC), TN, and TP were determined by external heating with potassium dichromate, Kjeldahl nitrogen determination, and molybdenum–antimony colorimetry, respectively (Liu et al. 2022; Wang et al. 2023a). Microbial biomass carbon (MBC), nitrogen (MBN), and phosphorus (MBP) were determined by the chloroform fumigation‐extraction method. Briefly, fresh soil samples were fumigated with chloroform and then extracted with 0.5 mol L^−1^ K_2_SO_4_ solution for the determination of MBC, followed by application of the Kjeldahl nitrogen method for the determination of MBN, in turn, by external heating with potassium dichromate; then, samples were extracted with 0.5 mol L^−1^ NaHCO_3_ solution for the determination of MBP by molybdenum‐blue colorimetry (Yuan et al. 2019; Xie et al. 2023).

### 2.5 Soil and Microbial Stoichiometric Ratios, Microbial Entropy, and Stoichiometric Imbalances

Soil and microbial biomass stoichiometric ratios were expressed as the ratios of C, N, and P contents. Microbial entropy (*q*_MB_) values were expressed as percentages of MBC, MBN, and MBP to SOC, TN, and TP (i.e., *q*_MBC_, *q*_MBN_, and *q*_MBP_) (Xie et al. 2023). The general formula is as follows:

(1) $$q_{\mathrm{MBC}}=\frac{\mathrm{MBC}}{\mathrm{SOC}}\times 100$$

(2) $$q_{\mathrm{MBN}}=\frac{\mathrm{MBN}}{\mathrm{TN}}\times 100$$

(3) $$q_{\mathrm{MBP}}=\frac{\mathrm{MBP}}{\mathrm{TP}}\times 100$$

Furthermore, following the approach of Yuan et al. (2019), we assessed the soil–microorganism stoichiometric imbalance according to the following equation.

(4) $$C_{\mathrm{imb}}:N_{\mathrm{imb}}=\frac{C:N}{\mathrm{MBC}:\mathrm{MBN}}$$

(5) $$C_{\mathrm{imb}}:P_{\mathrm{imb}}=\frac{C:P}{\mathrm{MBC}:\mathrm{MBP}}$$

(6) $$N_{\mathrm{imb}}:P_{\mathrm{imb}}=\frac{N:P}{\mathrm{MBN}:\mathrm{MBP}}$$

### 2.6 Statistical Analysis

Statistical analysis was performed using SPSS 25.0 software (IBM Corp., Armonk, NY, USA). Differences in soil nutrients, microbial biomass, microbial entropy, soil‐microbial stoichiometric ratios, and stoichiometric imbalance among treatments were tested by one‐way ANOVA, and the least significant difference (LSD) method was used to test whether the differences among treatments were significant (*p* < 0.05). The correlations between soil environmental factors, soil‐microbial stoichiometric ratios, microbial entropy, and stoichiometric imbalance at different N levels were analyzed using Origin 2022 software (OriginLab, Northampton, MA, USA). Furthermore, redundancy analysis (RDA) was conducted using Canoco 5.0 software (Microcomputer Power, Ithaca, NY, USA) to identify environmental factors that significantly influence soil *q*_MB_ and stoichiometric imbalance at different N levels. Figures and tables in this paper are expressed as mean ± standard error (SE) values (*n* = 5).

## 3 Results

### 3.1 Characterization of Changes in Soil Environmental Factors

**FIGURE 1:**
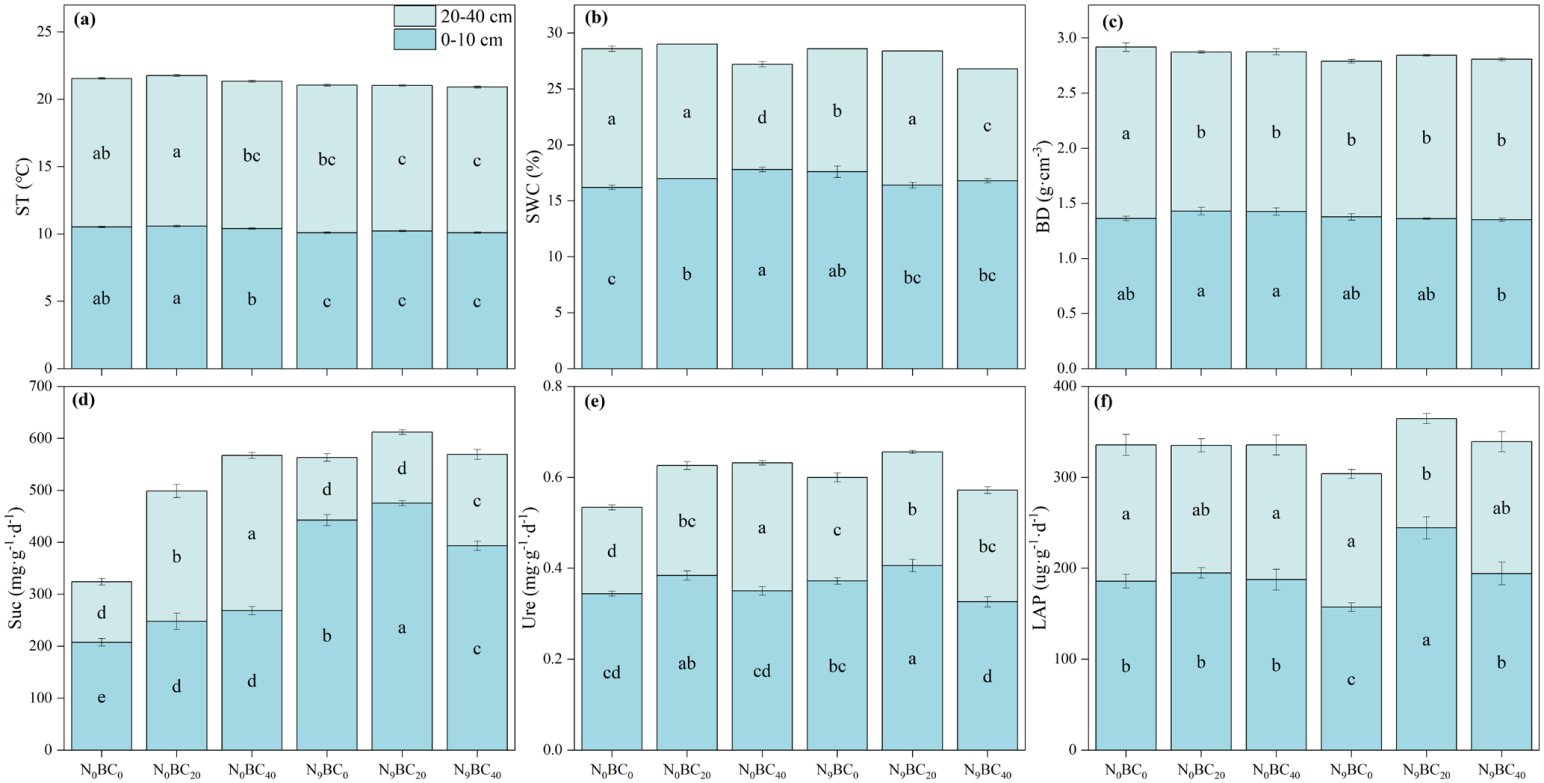
Changes in ST (a; soil temperature), SWC (b; soil water content), BD (c; bulk density), Suc (d; sucrase), Ure (e; urease), and LAP (f; alkaline phosphatase) contents in 0–40 cm soil of put‐down grassland under high N and BC additions. Lowercase letters in the graphs indicate significant differences (*p* < 0.05) between treatments under the same soil layer by LSD test.

Changes in ST, SWC, BD, Suc, Ure, and LAP contents in the two soil layers (0–20 cm, 20–40 cm) of the abandoned grassland differed under N and BC addition treatments (Figure 1a–f). N_9_ application generally decreased the 0–20 cm soil ST and LAP contents and increased the soil SWC and Suc contents, compared to the N_0_ control without additional N (*p* < 0.05). Meanwhile, both BC addition levels increased 0–40 cm soil Suc, Ure, and LAP content under N_0_ compared to no BC application (Figure 1d–f). Furthermore, there were significant differences in the effects of mixed additions of N_9_ and BC on 0–40 cm soil enzyme activities. Compared to the N_9_BC_0_ treatment, N_9_BC_20_ significantly increased 0–20 cm soil Suc, Ure, and LAP contents, but N_9_BC_40_ significantly decreased Suc and Ure contents (*p* < 0.05).

### 3.2 Soil C, N, and P Content and Related Chemometric Characteristics

**FIGURE 2:**
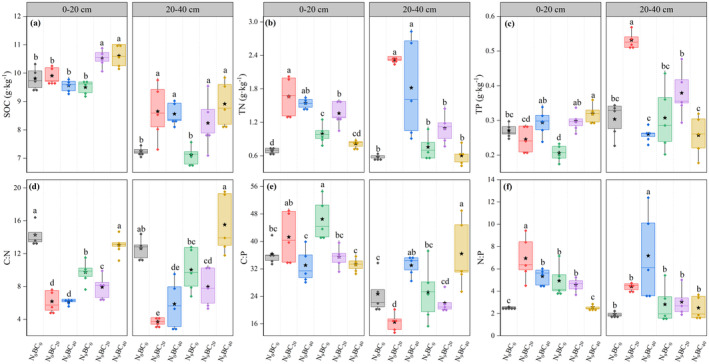
Changes in SOC (a), TN (b), and TP (c) contents and their stoichiometric ratios (d–f) in 0–40 cm soils of abandoned grassland under high N and BC additions. Box plots show mean (pentagram in box), median (solid line in box), and 75th and 25th percentiles (boundaries of box). Letters above the boxes indicate significant differences between treatments under the same soil layer by LSD test (*p* < 0.05).

Changes in SOC, TN, and TP contents and related stoichiometric ratios varied between soil layers (0–20 cm, 20–40 cm) of the abandoned grassland under N and BC additions (Figure 2). Under BC_0_ treatment, the effect of N_9_ addition on topsoil was more pronounced. Specifically, N_9_ addition significantly increased the values of TN (30.52%), C:P (27.93%), and N:P (93.80%) and decreased the values of TP (29.81%) and C:N (31.81%) in the 0–20 cm soil layer compared with N_0_ addition (*p* < 0.05). However, the effect on the SOC, TN, and TP contents and their stoichiometry in the 20–40 cm soil layer was not significant. Under N_0_, all BC addition levels in general increased the 0–40 cm soil SOC and TN contents and decreased the C:N values relative to treatments without BC application (Figure 2a,b,d). Among treatments, 0–40 cm soil SOC and TN contents were highest under BC_20_ addition, which was significantly higher (*p* < 0.05) than that under BC_0_ addition by 9.92% and 221.59%, respectively (Figure 2a–b). In addition, compared to the N_9_BC_0_ treatment, the N_9_BC_20_ treatment significantly increased the SOC in each soil layer (i.e., 0–40 cm) and the TN and TP content in the 0–20 cm soil layer and decreased the C:P and N:P values in the 0–20 cm soil layer (*p* < 0.05).

### 3.3 Soil MBC, MBN, MBP Contents, and Related Stoichiometric Ratios

**FIGURE 3:**
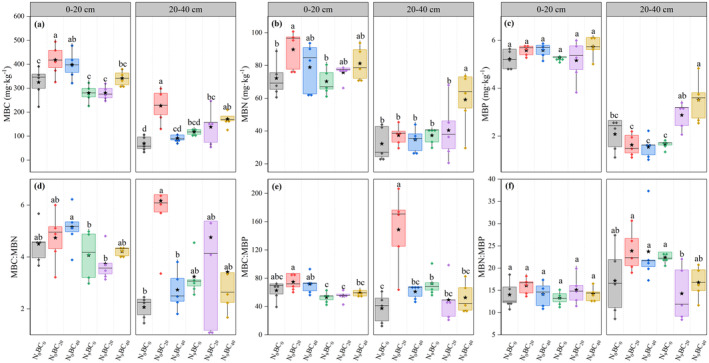
Changes in MBC (a), MBN (b), and MBP (c) contents and related stoichiometric ratios (d–f) in 0–40 cm soils of the abandoned grassland under high N and BC additions. Box plots show mean (pentagram in box), median (solid line in box), and 75th and 25th percentiles (boundaries of box). Letters above the boxes indicate significant differences between treatments under the same soil layer by LSD test (*p* < 0.05).

The effects of N_9_ addition on microbial biomass varied between the two soil layers under BC_0_ (Figure 3). Briefly, N_9_ addition generally reduced the values of MBC, MBN, and the stoichiometric ratio of 0–20 cm soil relative to N_0_, while the opposite trend was observed in the 20–40 cm soil layer. Under N_0_ and N_9_, all BC additions increased the 0–40 cm soil MBC, MBN, and MBP contents and microbial stoichiometric ratios compared to no BC application. Among treatments, under N_0_, the inclusion of BC_20_ led to a notable (*p* < 0.05) rise in MBC and MBN within the 0–40 cm soil layer, along with increases in the values of MBC:MBN and MBP within the 20–40 cm layer, in contrast to the effect of BC_0_ (Figure 3a,b,d,e). However, BC_40_ significantly (*p* < 0.05) increased MBN (58.48%) and MBP (111.30%) contents in the 20–40 cm soil layer under N_9_ addition.

### 3.4 Soil *q*_MB_ and Stoichiometric Imbalances

**FIGURE 4:**
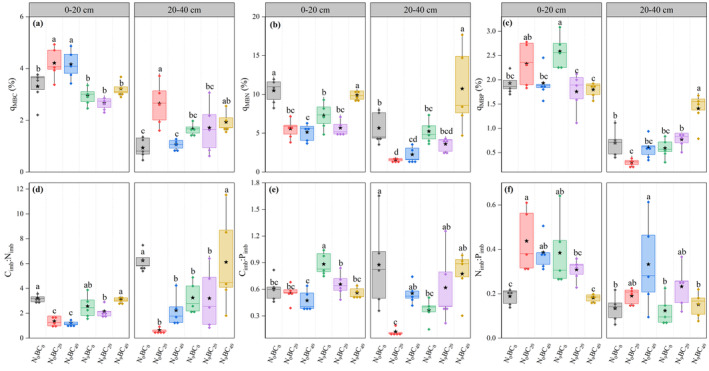
Changes in *q*_MB_ (a–c) contents and stoichiometric imbalances (d–f) in 0–40 cm soil of abandoned grassland under high N and BC additions. Box plots show mean (pentagram in box), median (solid line in box), and 75th and 25th percentiles (boundaries of box). Letters above the boxes indicate significant differences between treatments under the same soil layer by LSD test (*p* < 0.05).

Changes in *q*_MB_ content and stoichiometric imbalance values varied among soil layers of the abandoned grassland under N and BC additions (Figure 4). Under BC_0_, N_9_ addition significantly (*p* < 0.05) decreased the *q*_MBN_ values of 0–20 cm soil and C_imb_:N_imb_ and C_imb_:P_imb_ values of 20–40 cm soil compared to N_0_, while increasing the *q*_MBP_, C_imb_:P_imb_, and N_imb_:P_imb_ values of 0–20 cm soil. Under N_0_, all BC additions in general significantly (*p* < 0.05) increased *q*_MBC_ and N_imb_:P_imb_ values and decreased *q*_MBN_, C_imb_:N_imb_, and C_imb_:P_imb_ values in the 0–40 cm soil compared to no BC application (Figure 4a,b,d–f). In contrast, under N_9_ addition, C_imb_:P_imb_ and N_imb_:P_imb_ values of the 0–20 cm soil layer were gradually decreased as BC addition was increased (*p* < 0.05) (Figure 4e,f).

### 3.5 Relationship Between Soil Environmental Factors and *q*_MB_ and Stoichiometric Imbalances

**FIGURE 5:**
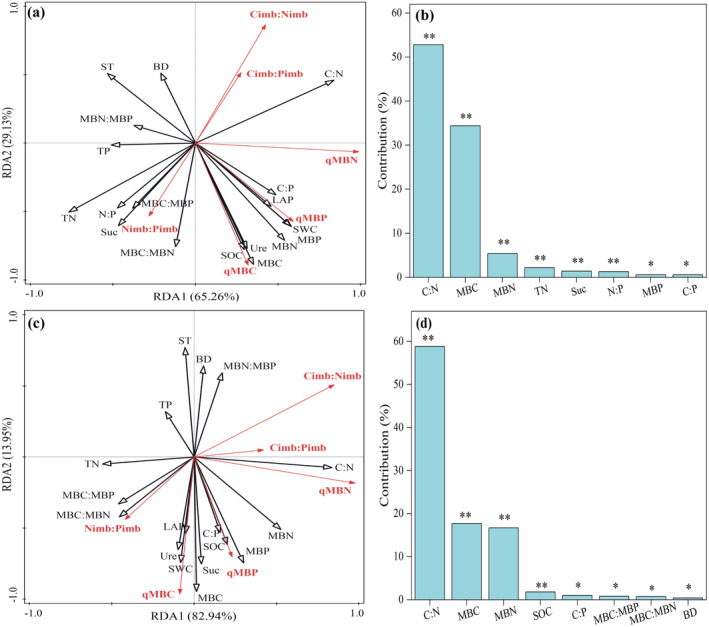
RDA was used to screen out the major factors that significantly influenced the changes in soil *q*_MB_ and stoichiometric imbalance under N_0_ (a, b) and N_9_ (c, d) additions. Asterisks indicate significant differences: *p < 0.05; **p < 0.01.

RDA showed that our selected environmental factors (i.e., soil physicochemical properties, enzyme activities, microbial biomass, and associated stoichiometric ratios) cumulatively explained 94.39% and 96.89% of the variation in *q*_MB_ and stoichiometric imbalance under N_0_ and N_9_ additions, respectively, successfully reflecting the variation in *q*_MB_ and stoichiometric imbalance (Figure 5a,c). Among measured environmental factors, C:N, MBC, MBN, and C:P were the main drivers that jointly influenced the changes in *q*_MB_ and stoichiometric imbalance under N_0_ and N_9_ additions, with total contributions of 93.20% and 94.20%, respectively (Figure 5).

**FIGURE 6:**
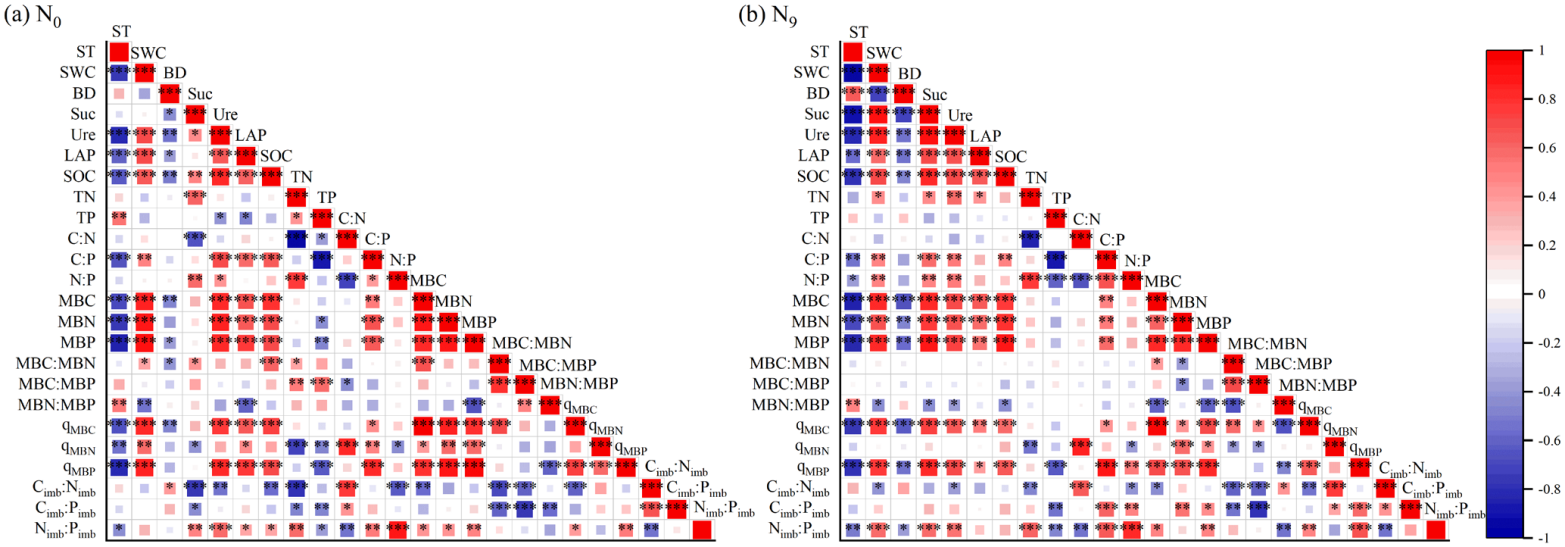
Heat map of correlation between soil microbial entropy, stoichiometric imbalance, and environmental factors under N_0_ (a) and N_9_ (b) addition. Progressively increasing * indicates that the correlation between the two factors is significant at the 0.05, 0.01, and 0.001 (*p*) levels, respectively. The various colors in the figure depict the relationship between the two factors, with the intensity of the colors reflecting the strength of the correlation coefficient.

There were also some differences in the factors affecting changes in soil *q*_MB_ and stoichiometric imbalance at different N levels. For example, soil TN, Suc, N:P, and MBP effectively explained the changes in soil stoichiometric imbalance under N_0_ (total contribution = 5.5%) (Figure 5b). Among these factors, negative significant correlations (*p* < 0.05) were found between TN, N:P, *q*_MBN_, and C_imb_:N_imb_ and positive significant relationships (*p* < 0.05) were found between TN, Suc, N:P, MBP, and N_imb_:P_imb_ (Figure 6a). Meanwhile, highly significant positive correlations (*p* < 0.001) were observed between soil *q*_MB_ and MBP (Figure 6a). However, SOC, MBC:MBN, MBC:MBP, and BD effectively explained the changes in soil *q*_MB_ and stoichiometric imbalance under N_9_ addition (total contribution = 3.7%) (Figure 5d). Among these factors, a highly significant positive correlation (*p* < 0.001) was observed between SOC and both *q*_MBC_ and *q*_MBP_ (Figure 6b). Additionally, significant negative correlations (*p* < 0.05) were found between MBC:MBN, MBC:MBP, *q*_MBN_, C_imb_:N_imb_, and C_imb_:P_imb_ (Figure 6b).

## 4 Discussion

### 4.1 Effect of High N and BC Additions on Soil Nutrients and Their Stoichiometric Ratios

Changes in soil C, N, and P contents and their related stoichiometric ratios are susceptible to the combined effects of external climate, management practices, and other factors (Yuan et al. 2019; Ren et al. 2024). Generally, owing to variation in topography, climate, weathering processes, vegetation cover, and microbial activity (Bargali et al. 2018), as well as several other biotic and abiotic factors (Bargali, Padalia, and Bargali 2019; Pandey et al. 2024), the physicochemical properties of soils vary spatially and temporally, and their responses to climate change also vary considerably (Gong, Zhang, and Guo 2019; Hu et al. 2023). Prior research has indicated that the effects of progressively higher N deposition on soil nutrient and stoichiometric ratios may increase, decrease, or exhibit (Gong, Zhang, and Guo 2019; Han et al. 2019; Hu et al. 2023). Mauro et al. (2021) suggested that ecosystem characteristics of different study sites (such as climate, vegetation characteristics, biological communities, and soil texture) are the key factors underlying such differences in findings.

Our present study indicated that high N addition had a more pronounced effect on nutrient and stoichiometric ratios in the surface soil, decreased surface TP and C:N values, and increased TN, C:P, and N:P values. Comparable findings were also documented by Gong, Zhang, and Guo (2019). Recent research has indicated that N addition can cause plant root systems to exhibit superficialization in the soil profile (Liu et al. 2022). The complex root structure and appropriate soil physical properties in the surface layer can cause the influence of external climate fluctuations on the topsoil to be more intense than in the deeper layers (Figure 1a–c) (Zhang, Lin, and Zeng 2024). Soil P elements were mainly derived from rock weathering and atmospheric P deposition; however, the process of parent material mineralization is slow, and atmospheric P deposition is relatively low in terrestrial ecosystems (Wang et al. 2023a). Therefore, only a small portion of the significant changes in soil P may be influenced by the rate of degradation of soil organic matter and the uptake and utilization of P by plants (Li et al. 2022; Wang et al. 2023a). In the present study, high N addition may have led to more soil P elements being utilized through promoting the growth of grasses (Gong, Zhang, and Guo 2019). Meanwhile, negative correlations were observed between soil TP and both C:P and N:P values under N_9_ addition (Figure 6b). This further suggests that excessive N addition may shift the soil from a state of N‐limitation to a state of P‐limitation, alleviate the inhibition of plant growth by soil N, reduce soil TP content, and increase soil C:P and N:P values by inhibiting LAP activity (Figure 1f). In addition, high N addition increased topsoil TN content. On the one hand, high N addition can induce compensatory soil N losses through pathways such as N_2_O emissions and NO_3_^−^ leaching, insufficient to fully offset the increase in N inputs, thus causing N accumulation (Toteva et al. 2024). Furthermore, in the present study, high N addition significantly reduced soil C:N values (Figure 2d). This indicated that the microbial demand for N was satisfied, which favored the microbial decomposition of apoplastic materials and led to an increase in the N content returned to the soil surface (Liu and Wang 2020; Li et al. 2022). Currently, the impact of N addition on SOC is highly contested. Previous reports have indicated that N addition contributes to SOC input and accumulation by promoting plant growth and increasing litter accumulation (Thakur et al. 2023; Zou et al. 2023). Certain studies indicate that N addition can exacerbate the leaching of organic matter from the soil, leading to the discharge of large amounts of C‐containing gases, which reduces SOC content (Zhang et al. 2019). However, our present research showed that the effect of high N addition on SOC content was not significant, consistent with the findings of Xiao et al. (2021b). Potential factors contributing to this debate may be related to the study area, the quantity of N applied, the time of N application, and soil texture. Moreover, the significantly lower C:N values observed under N_9_ addition showed a significant negative correlation with TN (Figure 6b), further indicating that N_9_ addition resulted in higher N input than C input and the relatively weak response of elemental C to high N addition.

We observed that BC_20_ significantly enhanced SOC, TN, and TP at the N_0_ level, but high BC addition (BC_40_) attenuated the promotion of soil nutrients by BC. Numerous studies have shown that BC is rich in nutrient elements and characterized by its loose and highly porous structure and associated strong adsorption (Yan et al. 2022). These characteristics enhance soil adsorption of the elements C, N, and P, increase the retention capacity of these soil elements, and increase the nutrient content of soil by reducing leaching losses (Khadem et al. 2021; Zhao et al. 2023). In addition, with BC_40_ addition in this study, the soil–microbe stoichiometric imbalance values were higher than those under BC_20_ addition (Figure 4d–f). This further indicates that excessive BC addition reduced the growth efficiency of soil microorganisms, which in turn attenuated the rate of nutrient accumulation in the soil. This is consistent with some previous studies showing that excessive BC addition can both disrupt the optimal environment and community structure for soil microbial growth and reduce the growth and reproduction rate of microorganisms (Zheng et al. 2018; Zhang et al. 2022c; Zou et al. 2023). Moreover, BC addition decreased soil C:N values and increased N:P values at the N_0_ level (Figure 2d,e). This demonstrated that BC addition alleviated the N‐limitation of the soil and improved the microbial decomposition capacity, and TN responded more strongly to BC addition. The significant relationship between C:N and N:P values and TN under N_0_ in this study also verified this observation (Figure 6a).

Notably, compared to high N addition alone, the combined application of both high N and BC increased the 0–40 cm soil SOC and TP content, whereas the TN content was increased under the mixture of low BC and high N addition, which is similar to findings by Thakur et al. (2023). The reasons for this phenomenon may be multifaceted. On the one hand, the relatively unstable C of BC itself could have been gradually decomposed in the presence of sufficient N to replenish soil nutrients (Xu et al. 2022; Zhang et al. 2022a). Meanwhile, BC promoted the formation of organic‐mineral complexes during its retention in the soil and stimulated the activities of Suc and LAP in the soil under the stimulatory effect of N application (Figure 1d,f), thus increasing the accumulation of C and P elements in the soil (Zhang et al. 2022a). It has been demonstrated in many studies that N in BC mainly occurs in the form of heterocyclic aromatic N, which cannot directly and effectively supplement the soil with elemental N (Xu et al. 2022; Thakur et al. 2023). However, the addition of BC can promote plant root proliferation and increase the nutrient uptake capacity of the root system (Yu et al. 2016). The addition of low amounts of BC and high N can alleviate the limitation of plant growth by soil elements and increase continued higher soil TN content by increasing external N inputs such as the amount of plant litter (Gong, Zhang, and Guo 2019; Toteva et al. 2024). However, high C provided by high BC addition can lead to competitive uptake of N by plants and microorganisms (Liu et al. 2024). Furthermore, high amounts of BC significantly reduced surface soil BD under mixed application with high N (Figure 1c). This would be expected to increase the emissions of N‐containing gases from the soil, offsetting the input of elemental N, which in turn reduced the TN content and moderated the observed changes in C:N and N:P values (Figure 6b) (Liu et al. 2024).

### 4.2 Effect of High N and BC Additions on Soil Microbial Biomass and Their Stoichiometric Ratios

Previous reports indicated that microbial biomass reflects the status of soil nutrient pool sources, and variations in their stoichiometric ratios can indicate shifts in the structure of the microbial community (Cao et al. 2019; Dong et al. 2022). A recent study indicated that the observed soil microbial community exhibited a dominant bacterial population when the MBC:MBN ratio ranged from 3 to 6 and a dominant fungal population when it was greater than 6 (Xie et al. 2023). In the present study, the mean value of MBC:MBN under different N and BC addition levels was 4.07, indicating that soil bacteria were the dominant microbial population at the present study site. Meanwhile, the soil MBC:MBP (mean 66.67) value in the study area was lower than the national mean soil MBC:MBP value (70.20), while the MBN:MBP (mean 17.10) value was higher than the globally reported soil mean (6.90) (Cao et al. 2019; Li, Yu, and Song 2022). This implies that soil microorganisms in the study area have relatively higher N effectiveness and lower P effectiveness under different N and BC additions and that soil microbial activities may be P‐limited (Cao et al. 2019).

Our study observed that BC_20_ addition effectively increased soil microbial biomass and its ratio at the N_0_ level, but high BC addition attenuated the promotion effect of BC. This may be because the porosity and abundant nutrients of BC itself provide a safe and appropriate habitat as well as sufficient energy substrate for microbes to grow and reproduce (Figure 2a–c), thus promoting the accumulation of microbial biomass (Liu et al. 2024). Meanwhile, the significantly higher soil MBC:MBN and MBC:MBP values at the N_0_ level (Figure 6a) illustrated that BC promoted MBC more than MBN and MBP. However, excessive BC addition may have excessively elevated the soil water content (Figure 1b) and thus disrupted the optimal environment and community structure for the growth of soil microorganisms by affecting soil aeration, which in turn reduced the growth and reproduction rate of some soil microorganisms (Zheng et al. 2018; Zou et al. 2023).

Previous reports suggested that N addition could cause soil acidification and reduce microbial biomass by lowering alkali cations in the soil, causing the death of microorganisms that are sensitive to and intolerant of high N (Li et al. 2021a; Qin et al. 2024). Nevertheless, our findings indicated that high N addition by itself did not have a significant impact on the soil microbial biomass. This may be related to both the sensitivity of soil microbial biomass to N addition in different regions and the short duration of N application in this study. Notably, similar to the results reported by Hu et al. (2023), the combined addition of high N and BC reduced the MBC content of the topsoil compared to the addition of BC alone. Numerous scholars have suggested that the factors influencing the reduction of soil MBC under the combined addition of high N and BC may be multifaceted (Sun et al. 2022). Firstly, high BC input has a negative exponential effect on soil C mineralization, and high N reduces the co‐metabolism of degradable compounds and C on the BC surface, thus inhibiting increases in elemental C in microorganisms (Zhang et al. 2022a). Secondly, high N reacts with potentially toxic compounds (e.g., heavy metals, etc.) and phenolic compounds in BC and stimulates the formation of more filamentous attachments on the BC surface, which adsorb and utilize elemental C secreted by the plant surface root system (Oladele et al. 2019). Furthermore, high N stimulated the adsorption of dissolved organic carbon by BC, which promoted the conversion of SOC to dissolved matter and the release of elemental C to the outside world, which in turn reduced the conversion of SOC to MBC (Zhang et al. 2019, 2022a). Finally, high N diminishes the positive effects of BC on soil microorganisms by significantly increasing soil C_imb_:N_imb_ values (Figure 4d), which resulted in an imbalance in the nutrients required for microbial growth and reduced the efficiency of microbial C assimilation by reducing bacterial diversity (Li et al. 2021b; Sun et al. 2022). However, it is important to highlight that the combined addition of high N and BC increased soil MBN and MBP content in the subsoil (20–40 cm) compared to high N additions alone. Previous reports indicated that short‐term N additions altered the microbial activity and community structure composition of soils mainly by affecting soil acidification status and soil basal nutrient effectiveness, which in turn were more pronounced in the surface layer (Oladele et al. 2019; Xia et al. 2020). This may have caused the deeper soils in the study area to be mainly affected by BC at high N and BC combined additions. This was attributed to the fact that high N promoted the structural degradation rate of BC, which led to the migration of BC into the deeper layers of the soil, thereby satisfying the demand of the subsoil microorganisms for nutrient substrates and increasing the content of soil MBN and MBP (Xia et al. 2020). Furthermore, the combined addition of high N and BC resulted in significantly lower subsoil MBC:MBP and MBN:MBP values (Figure 3e,f). This further indicates that high N and BC additions increased the buildup of MBN and MBP in the lower soil layers, and alleviated the limitation of N and P availability during the growth of microorganisms (Cao et al. 2019).

### 4.3 Effect of High N and BC Additions on *q*_MB_ and Stoichiometric Imbalances

The value of *q*_MB_ is controlled by the amount of soil substrate, and its changes effectively reflect the trend in resource effectiveness, which in turn can predict the subtle changes of soil microorganisms under external environmental changes (Xie et al. 2023). Our findings revealed that the N_9_ addition decreased topsoil *q*_MBN_ values and increased *q*_MBP_ values. This suggests that a high level of N addition influenced the stability of topsoil N and P pools and inhibited and promoted microbial activities used for N and P cycling, respectively, to some extent. The effect of high N addition on TN, TP, C:N, and C:P in topsoil in this study (Figure 2b–e) further supports this view. Previous reports indicated that, according to the stoichiometric limitation theory, soil microorganisms preferentially take up the most suitable elemental components, which in turn regulates the imbalance between the stoichiometry of resource supply and microbial demand (Chi et al. 2023; Xie et al. 2023). Meanwhile, higher imbalance values indicate the low quality of soil resources and decreased growth efficiency of microorganisms (Yuan et al. 2019). We found that high N addition decreased topsoil C_imb_:N_imb_ values and increased C_imb_:P_imb_ and N_imb_:P_imb_ values. On the one hand, according to the RDA results, C:N and C:P were the main drivers that jointly affected the observed change in the stoichiometric imbalance (Figure 5). However, high N addition reduced soil microbial growth efficiency and nutrient conversion to microbial biomass to some extent by positively affecting C:P and N:P and negatively affecting soil C:N (Figure 2d–f). Furthermore, high N addition induced the plant root system to exhibit shallow stratification and led to the imbalance of elements by disrupting the balance between elemental inputs and losses, which then inhibited the growth of soil microorganisms and biomass accumulation (Xie et al. 2023).

We found that BC addition alone effectively increased soil *q*_MBC_ and N_imb_:P_imb_ values and decreased *q*_MBN_, C_imb_:N_imb_, and C_imb_:P_imb_ values at the N_0_ level. This indicated that BC addition alone improved the efficiency of microbial utilization of soil C resources. The unique structure and abundant nutrient elements of BC can alter the accumulation of soil microbial biomass (Liu et al. 2024). Meanwhile, MBC and MBN were important drivers influencing microbial entropy changes (Figure 5a–b), and the effects of BC on MBC and TN were stronger. This also led to BC addition alone promoting the conversion of SOC to MBC and decreasing the conversion of TN to MBN, which resulted in changes in soil microbial entropy and stoichiometric disequilibrium. In addition, the combination of both high N and BC significantly reduced the surface soil *q*_MBP_, C_imb_:P_imb_, and N_imb_:P_imb_ values compared to high N addition alone. This suggests that BC addition altered the effect of high N on soil microorganisms and improved the utilization efficiency of soil C and N resources by reducing the microbial P assimilation efficiency (Li et al. 2021b; Sun et al. 2022), thus altering the response of soil microbial entropy (Figure 3a–c). Meanwhile, different levels of correlation were observed between both soil–microbial stoichiometric imbalance and soil–microbial biomass and their stoichiometric ratios at the N_0_ and N_9_ levels (Figures 5 and 6). This further suggests that high N and BC additions, by affecting soil nutrient inputs, allow soil microorganisms to adapt to changes in soil–microbial stoichiometric imbalance caused by the external environment by regulating their biomass (Figure 4).

## 5 Conclusion

Our short‐term experiments showed that high N and BC additions altered the stoichiometric patterns of soil–microbe interactions in the present Loess Plateau grassland study site. Among the treatments, high N addition had a stronger effect on the stoichiometric stability of N and P pools in the surface soil–microbial interactions, inducing a decrease in *q*_MBN_ and C_imb_:N_imb_ values and an increase in *q*_MBP_, C_imb_:P_imb_ and N_imb_:P_imb_ values in the surface soil. Meanwhile, BC addition had a more pronounced effect on TN and MBC, relieving soil limitation of N elements and increasing the efficiency of microbial utilization of C resources. In addition, BC addition significantly increased the level of soil nutrients and microbial biomass, but the positive effect of BC on soil was weakened when the BC application rate was too high. Thus, BC addition was able to reduce the negative effects of high N on soil nutrients and microbial biomass, improve the utilization efficiency of soil for N resources, and change the soil–microorganism stoichiometry. However, long‐term field observations should be conducted to avoid uncertainty of the results of short‐term experiments.

## Author Contributions

**Shuainan Liu:** conceptualization (lead), formal analysis (equal), investigation (equal), methodology (equal), writing – original draft (lead). **Mingjun Xie:** data curation (equal), formal analysis (equal), methodology (equal). **Wende Lu:** formal analysis (equal), methodology (equal). **Xinyue Zhang:** formal analysis (equal), methodology (equal). **Mengyin Du:** investigation (equal), methodology (equal). **Yao Yao:** investigation (equal), methodology (equal). **Jianyu Yuan:** investigation (equal), methodology (equal). **Guang Li:** funding acquisition (equal), supervision (equal), writing – review and editing (equal).

## Conflicts of Interest

The authors declare no conflicts of interest.

## Data Availability

The soil nutrient, microbial biomass, and enzyme activity data are available on Dryad at https://doi.org/10.5061/dryad.ncjsxkt48 (https://datadryad.org/stash/share/WDUpZJTIvDd3wqjY_lwR4uoM4b5z80KZolchKOvUubo).
